# Harnessing the Power of Collaboration to Expand the Coverage and Equity of COVID-19 Vaccinations in India: A Community Collaboration Model

**DOI:** 10.3390/vaccines11061022

**Published:** 2023-05-24

**Authors:** Gopal Krishna Soni, Surbhi Seth, Sonal Arora, Kapil Singh, Amrita Kumari, Natasha Kanagat, Rebecca Fields

**Affiliations:** 1 John Snow India Pvt. Ltd. (JSIPL), Plot No. 5 & 6, First Floor Allied House, Pocket 10, Sector B, Vasant Kunj, Delhi 110070, India; 2 World Health Organization (WHO), Nirman Bhawan Maulana Azad Road, Delhi 110011, India; 3 John Snow Inc., 2733 Crystal Drive, 4th Floor, Arlington, VA 22202, USA

**Keywords:** COVID-19 vaccine, inequities, localization, engagement strategies, collaboration, NGO, community engagement, vulnerable and at-risk

## Abstract

Early in 2021, India embarked on the uphill journey of the COVID-19 vaccination of the largest population group in the world in a prioritized manner and in the shortest possible time. Considering the endless variety of geography and diverse socio-economic demographic, religious, and community contexts, there was a high likelihood of certain population subgroups with known vulnerabilities facing inequities, which were anticipated to be further accentuated by a digital divide. This necessitated devising solutions for such communities in a localized manner to aid the local government in breaking the service access and uptake barriers with an inclusive approach. To bridge this vital gap, the Momentum Routine Immunization Transformation and Equity project implemented a three-tiered collaboration, viz., government, non-governmental organizations (NGOs), and a wide range of vulnerable and at-risk communities, utilizing knowledge exchange and use of data. The project implemented localization strategies through the NGOs for community engagement in conjunction with government vaccination teams to universalize COVID-19 vaccination uptake up to the last mile. The collaboration resulted in reaching close to 50 million beneficiaries through messaging and facilitated the administration of more than 14 million vaccine doses, including 6.1 million doses for vulnerable and marginalized communities in 18 States and Union territories in India, along with suggesting implications for public health practice and research.

## 1. Introduction

The Indian Government launched its COVID-19 vaccination drive in January 2021, with the objective of vaccinating the world’s largest national population group of 1.06 billion people [1]. Using a phased roll-out approach, the vaccination program prioritized individuals with the highest risk of infection, such as health personnel, frontline workers, the elderly (those over 60 years of age), and people with comorbidities. Gradually, the immunization scope was expanded to include the general population, including children above 12 years [2]. The vaccination program was guided by the “National Expert Group on Vaccine Administration for COVID-19” (NEGVAC), (constituted in August 2020), to formulate a comprehensive action plan for vaccine administration in India [3]. Within 6 months of the launch of the COVID-19 vaccination program, the country encountered the second wave of the COVID-19 pandemic. The Delta variant, prevalent during this wave, affected the country disastrously, spreading in the high population-density areas of the country [4]. This wave (April–September 2021), left health functionaries overwhelmed by the tasks of mitigating the adverse impact of the disease and implementing infection management mandates [5]. There was widespread vaccine hesitancy due to misinformation, especially in rural areas [6], encompassing nearly 65% of the Indian population [7]. Based on previous experiences from routine immunization with respect to vaccine hesitancy and geographical inaccessibility, the Government of India and partnering organizations were convinced that growing resistance towards the COVID-19 vaccine could not be overcome effectively with the current system in place, and the marginalized and vulnerable population groups could not be focused on properly in the current system [8,9].

According to the ‘UN Women,’ adolescents, teenagers, children, women, the elderly, people with disabilities, indigenous people, refugees, migrants, and minorities face the highest social and economic marginalization [10]. Marginalized groups are generally difficult to reach and are vulnerable. Social and religious norms, geographical constraints, transient movement due to nomadic lifestyles, discrimination by society, and insecurity are some obstacles and impediments connected to COVID-19 vaccination faced by the marginalized and vulnerable. The importance of effective vaccine distribution planning and transparency in identifying marginalized populations has been emphasized [11]. Ensuring that COVID-19 vaccinations are disseminated equitably and are widely available, regardless of socioeconomic class or geographic location, is critical. Challenges such as supply–demand discrepancy (shortage of doses), vaccine hesitancy (due to cultural or faith-based biases), the digital divide (inability to access the digital application for vaccination registration), and the lack of access in remote areas and for vulnerable populations (such as the elderly, the differently-abled, prison inmates and truckers) call for innovative solutions [12]. Available approaches used in effective vaccine equity strategies include expanding vaccine production and distribution, prioritizing disadvantaged people while administering vaccination, utilizing community-based collaborations, etc. [13]. To attain high levels of coverage, the literature emphasizes various vaccine distribution approaches (e.g., utilizing outdoor spaces and pharmacies) and communication methods, as well as catch-up programs such as setting up outdoor and drive-through vaccination clinics [14]. Addressing vaccination hesitancy requires raising awareness and giving accurate information regarding the safety and efficacy of COVID-19 vaccines. With these methods of vaccine delivery and demand generation, a multifaceted and collaborative approach becomes necessary.

Collaboration is integral to the success of any public health program. The fundamental assumption underlying collaborative participation is that addressing complex problems involves a combination of diverse resources owned or managed by different organizations [15]. Collaborations may demand sustained commitment from those involved to benefit from partner loyalty, accountability, and an enhanced feeling of ownership. Moreover, they create a system ready for future initiatives. While resource sharing is not a new concept, it is more challenging in the context of COVID-19 vaccination due to scale, time shortage, and the demand–supply gap. Hence, strategic alignment in collaborations might be imperative for resource sharing when vaccinating people in India’s diverse cohort.

In August 2021, the MOMENTUM Routine Immunization Transformation and Equity (M-RITE) project, supported by the U.S. Agency for International Development (USAID) and led by John Snow India Pvt. Ltd. under the guidance of the Ministry of Health and Family Welfare (MoHFW) of the Government of India, pioneered multiple pathways for scaling solutions for reaching the most vulnerable with COVID-19 vaccination—particularly through successful collaboration with the community-oriented non-governmental organizations (NGOs), community influencers, religious leaders, and associations of people. The model presented in this paper introduces a novel case of innovation in collaborative management with a strategic focus on vulnerable and at-risk communities through collaborations with local NGOs. The learning and experiences shared in this paper provide considerations for public health managers to work beyond the boundaries of specialization and management style in forming and convening collaborations and providing a framework applicable to emergency and routine health scenarios.

## 2. Approach for Collaborative Participation

World Health Organization (WHO), in its operational planning guidelines to support country-preparedness and response to COVID-19, has prioritized risk communication and community engagement by recommending collaborating with civil society as an important approach to dealing with the impact of the pandemic [16]. Similarly, USAID has also prioritized COVID-19 vaccination in the most vulnerable population groups; reaching them could be more successful through collaboration [17]. Collaborations are categorized in public health literature as level-based (person, population, or research), solution-based, or third-party-based [18]. ‘Solution-based collaborations’ are geared toward specific problems such as diseases or outbreak conditions and have been utilized well in managing situations in the past [19,20]. The project’s multilevel collaboration is ‘solution-based’ as it engages ground-level NGOs in a sustainable network and strives to strengthen the skills of collaboration partners, as well as optimizing the use of limited resources to attain the common objective of equitable vaccination.

An extensive literature search for possible collaboration approaches did not yield results for a comprehensive collaborative approach suitable for vaccine delivery in emergency situations like COVID-19, but did provide insights for developing a collaborative model. For example, Solana EF’s public–private not-for-profit partnerships (PPNP) model (2014) suggests equitable allocation of tasks among partners to maximize value generation [21]. Gómez JJ (2004) advises that public health professionals strive to create long-term relationships with NGOs and incorporate information shared by NGOs into policy and program implementation [22]. He defines collaborative associations as partnerships with common aims, mutual respect, and access to information and decision-making procedures, as well as the partners’ accountability to their constituents. Gomez recommends supplementing rather than replacing government activity. In agreement with his recommendation, the project team was tasked with supporting the government’s vaccination efforts to ensure alignment with the Government, working on the implementation with project partners and NGOs in order to reach common objectives. The learnings from the preceding models were combined with the project team’s on-the-ground experience to offer the strategic collaboration model explained further in the paper.

**Figure 1:**
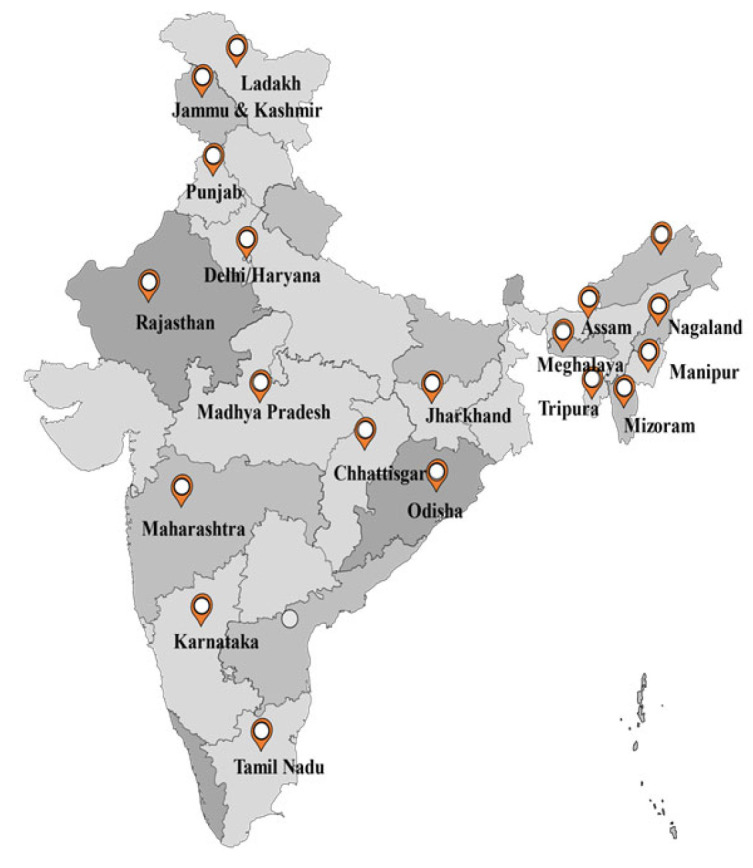
Project intervention states/UTs for collaboration.

The project team assisted the MoHFW of India at the national, state, and district levels to develop a collaboration mechanism that utilized the strengths of NGOs specializing in working with vulnerable and marginalized populations in 18 states and union territories (UTs) and 298 districts, as shown in Figure 1.

The collaboration followed a four-step consultative process between the government, donors, and project implementation team:

Step 1: Need-assessment for identification of areas of collaborative support and geographies for intervention.

The project team underwent multiple rounds of discussions with the MoHFW of India, as well as the project funders, to finalize the areas of intervention as indicated below:

- Technical assistance (TA) for vaccine planning and prioritization for equitable delivery, including supply chain and vaccine safety;
- TA for catalyzing service delivery and use, including innovative delivery modalities, health workforce expansion, capacity building, community engagement, demand generation, and risk communication;
- Identification of intervention geographies for addressing gaps in last-mile delivery based on the COVID-19 vaccination coverage up to August 2021. Eighteen states, including seven in the northeastern part of India, known for its hard-to-reach terrain, were identified for technical support and on-the-ground interventions;
- Concluding that the high-priority vulnerable populations, as the key target group, included, but were not limited to, the elderly age group, differently-abled people, remote and/or tribal communities, truckers, and migrants. Priority groups also included pregnant and lactating women, transgender people, people with disabilities, adolescents in childcare institutions, etc.

A detailed account of activities under each intervention area has been discussed at length in the forthcoming sections.

Step 2: Identification, onboarding, and capacity building of NGOs to address last-mile delivery gaps.

The project collaborated with NGOs, which had a significant role in implementation, particularly at the district level and below, to support last-mile service delivery and social mobilization and community engagement activities for equitable vaccine coverage. The selection criteria included: local NGOs (not international NGOs); active presence in the low-vaccine-coverage districts; valid Foreign Contribution Regulation Act (FCRA) certification; prior experience of working in USAID projects; understanding of local issues; the organization’s vision being aimed towards marginalized and vulnerable populations in the area; and strong history of financial management, administration, and compliance with laws.

Step 3: Devising risk-communication strategies for effective community-based collaboration with the flexibility to customize demand-generation activities.

The on-the-ground communication strategy was designed in accordance with the national government’s COVID-19 Vaccine Communication Strategy, and focused on managing vaccine eagerness, hesitancy, and crises with respect to fear of adverse events following immunization. The project team supported state and district governments with demand-generation activities for cohorts for social mobilization and increasing awareness across project states and districts.

The team emphasized collaboration with the local governments, as well as in workplaces, community-based organizations (CBOs), and faith-based organizations (FBOs) to customize interventions to meet the objectives of demand generation and target and reach specific population groups (e.g., truckers, immigrants, older adults, pregnant and breastfeeding women, and differently-abled people, among others). Activities included mapping and reaching out to vulnerable populations, raising awareness, mobilizing communities, and connecting beneficiaries to the health system. Aligning with the national and state government needs, the NGOs customized the delivery of messages during the ‘Har Ghar Dastak’ (knock every door) campaign of the national government.

Step 4: Devising a monitoring, evaluation, and learning (MEL) framework for reporting collaborative efforts.

The project identified requirements for project monitoring, evaluation, and learning for the data collection and management system, and developed a system, in collaboration with the USAID Strategic Information Team, to ensure maximum efficiency in data reporting.

The team also developed standardized data-capturing and reporting formats for NGOs. To ensure the quality of data reported by the NGOs, the project state teams and supervisory-level staff of the NGOs collated details of beneficiaries from the reported line lists and checked the status of vaccination of the beneficiaries (through field visits or telephone calls). Around 5% of reported vaccinations were subject to back-checks on a monthly basis.

## 3. Implementation

**Figure 2:**
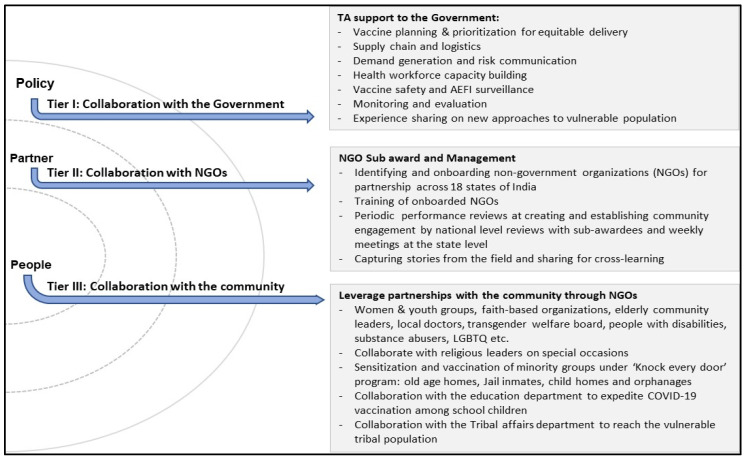
Tiered collaboration process.

The collaboration model was designed and implemented with a three-tiered approach, viz., collaboration with the government, NGOs, and the community, as depicted in Figure 2.

### 3.1. Collaboration with the Government

The project’s efforts were complementary to the efforts of the national and state governments in multiple ways, such as the provision of technical assistance, catalyzing program implementation, monitoring, and evaluation, and knowledge exchange.

Technical assistance: The project team collaborated with the national government on capacity-building, vaccine supply-chain management, data analysis of vaccination coverage, communication strategies, adverse events following immunization (AEFI), and most importantly, designing various vaccine delivery approaches. Support was also seconded to the Government Medical Store Departments (GMSD) to facilitate a regular and uninterrupted supply of vaccines and syringes, and the tracking of vaccines from manufacturers to regional and state stores in coordination with supply-chain personnel. On many occasions, the collaboration allowed the generation of crucial evidence, such as shortages of vaccines, hesitancy issues, expiring vaccines, etc., which led to the re-alignment of efforts that enhanced the vaccination program. This was especially important when the vaccination staff and healthcare workers were overwhelmed in mitigating the effects of the second COVID-19 wave. Notably, the project team advocated for and facilitated the redistribution of near-expiry vials to districts with low coverage.

Catalyzing project implementation: The project teams at the sub-national level identified barriers to vaccination service uptake and demand, and concurrently provided information to district administrations to advocate for organizing special camps tailored to their community’s needs. One such example of facilitating vaccination among daily wage workers is shared in Box 1. Daily wage workers, including agricultural laborers, coal mine workers, and skilled and unskilled laborers, constitute 35.9% of the labor force in India [23]. They are constrained by long working hours, as well as wage loss if they miss a workday. Such barriers were related to vaccine inequity among diverse populations in other countries as well [24]. To ensure the vaccination of daily wagers, the project team made special efforts to include them in the COVID-19 vaccination drive.

Box 1 **Night camps for daily wagers.**

The **state of Jammu & Kashmir** is home to many migrant laborers. Health staff observed that it was difficult to vaccinate them during the working hours of the day. The project team, along with the sub-awardee, organized a rapid coverage survey, followed by an awareness drive and vaccination camp lasting three days, and running into the late evenings. Night camps were organized at three different sites, which resulted in the successful facilitation of vaccine doses at times convenient to the community.

In the **state of Maharashtra**, the tribal population of Nandurbar (district) is occupied in daily wage work. Due to the workers’ busy daytime schedules, they were unable to make time for vaccination. Successful advocacy by the project team with the district administration led to the organizing of night awareness-and-vaccination camps in the tribal settlements, resulting in outreach to 15,000 people with messages and nearly 1450 people with vaccination.

Similar initiatives were taken in other states such as **Punjab, Rajasthan**, and **Chhattisgarhx**.

Monitoring & evaluation: The state project teams supported government officials with their data management systems to ensure consistent data collection, systematic data cleaning, and sufficient analytics. Using the CO-WIN data portal which has been developed by the Government of India, the project team supported state governments with management information system (MIS) based factsheets for better decision-making, informing strategies to address low vaccine coverage and assessing the effectiveness of NGO-led interventions.

**Figure 3:**
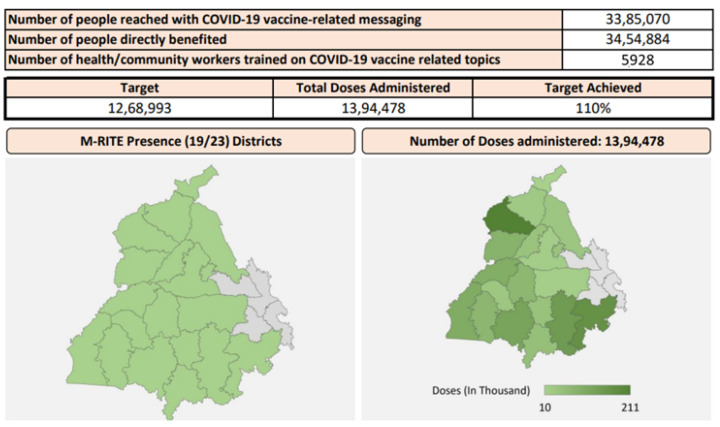
A glimpse of state fact sheets.

The state dashboards were automated for all 18 project states; the one presented in Figure 3 belongs to the state of Punjab. The dashboard is dynamic and has the functionality of representing performance on a weekly basis. It presents district-wise performance, by dose, by period, and by age group, and employs various data visualization tools. With this tool, data could be used to drive program strategy and decision-making and ensure a uniform understanding of the monitoring, evaluation, and learning systems and processes among the state and district teams and NGOs.

Knowledge sharing is imperative to collaboration. For uninterrupted functioning of the project collaboration, all partners under the project met with donors at the national level each month to discuss synergy in all critical areas of work. The project also conducted reviews/knowledge-sharing meetings with the NGOs and government at the sub-national level to share best practices and key learnings on the engagement of marginalized and vulnerable groups at the field level and share the support received from the local administration in all project regions, especially hard-to-reach areas, for vaccine supply.

### 3.2. Collaboration with NGOs

To increase the demand for, distribution of, and uptake of COVID-19 vaccination, particularly among vulnerable and marginalized populations, it was important to build public trust in vaccines and seek public participation. The project team, therefore, identified NGOs for partnership in districts that had low COVID-19 vaccination coverage in discussion with the state governments and were highly ranked in the priority list of state governments. The main objective of the collaboration was to identify low coverage and/or inaccessible areas; engage in the prioritization of these areas; and support vaccine delivery planning and supply-side interventions, demand generation through community engagement, and social mobilization. The project team and collaborating NGOs joined hands to support the physical mobilization of the elderly and persons with disabilities to vaccination centers; establish referrals, link-up beneficiaries for enhancing vaccine access and conducting follow-ups, and conduct need-based assessments to understand misconceptions regarding COVID-19 vaccination and combat vaccine hesitancy. The state project teams facilitated training for the onboarded NGOs’ human resources on project implementation activities. For effective counseling and outreach efforts, a detailed set of reference materials, including interpersonal communication (IPC) for rapport-building and frequently asked questions (FAQs) were used to allay fears and mitigate negative messaging in the community. The NGOs brought different forms of expertise and helped reach various marginalized and neglected groups, as depicted in Box 2.

Box 2 **Collaboration with NGOs with specific expertise.**

The project team collaborated with ground-based NGOs with diverse areas of specialization to mitigate the varied set of project challenges:

**Church’s Auxiliary for Social Action (CASA)**, an organization specialized in working with hard-to-reach communities in the northeastern region of India, has a strong presence among the faith-based community, and works closely with them to address common misconceptions and/or misinformation which cause vaccine hesitancy.

**HelpAge India**, with its expertise in providing elderly care, aided the project in overcoming resistance, mobility restrictions, and the digital divide (technological challenges) among the elderly.

**Samarthan-Centre for Development Support**, with its deep-rooted presence in tribal populations in Chhattisgarh, helped build trust in COVID-19 vaccination among hard-to-reach populations.

**Shanta Memorial Rehabilitation Centre (SMRC)** worked strongly to implement the “Near to home vaccination” advisory by MoHFW for all individuals who were unable to leave their homes for a variety of reasons, including illness and disabilities.

**
Transport Corporation of India Foundation (TCIF) supported** the vaccination of the trucker community and allied populations.

### 3.3. Collaboration with the Community

Gilmore et al. in 2020 [25] identified six main community-engagement actors in their evidence-based literature review: local leaders, community and faith-based organizations, community groups, health facility committees, individuals, and key stakeholders. These actors work by providing different functions: design and planning, community entry and trust building, social- and behavior-change communication, risk communication, surveillance and tracing, and logistics and administration. Working on the same principle, the project continues its engagement with major CBOs and FBOsin various states under the leadership of the respective state governments. Box 3 describes examples of how the cooperation of religious leaders generates vaccine confidence and demand among minority sub-groups.

Box 3 **Involving religious leaders from different geographies.**

**North India**: The project team worked closely with the Sikh Gurudwara Prabandhak Samiti (SGPC) in the state of Punjab. It conducted awareness generation activities and beneficiary counseling, shared a written appeal in favor of COVID-19 vaccination, and facilitated regular announcements from gurudwaras, which helped build vaccine confidence and enhance community engagement among the population of the state. The team also facilitated vaccination near the places of worship.

**Central India**: The project team worked closely with religious and community leaders of Tajjul Masjid (one of the biggest mosques in Asia), which is located in Bhopal, the capital city of Madhya Pradesh. The project team, under the leadership of the state government, sensitized and facilitated vaccination for around 400 religious leaders from various parts of the state, who in turn pledged to support COVID-19 vaccination from various mosques in the state. As a result of sensitization, the Masjid undertook intensive community-engagement activities to spread awareness, such as sharing messages through amplified announcements and local television.

**North East India:** In northeastern states, the project team collaborated with churches to create awareness among and mobilize people for vaccination by organizing vaccination camps on church premises, which helped to build vaccine confidence among the masses. The religious leaders were facilitated with vaccination on a priority basis so they could motivate community members to become vaccinated.

The strategy adopted by the project team was guided by inclusivity’, and ‘equity’, and was well-aligned with the government’s strategy for COVID-19 vaccination. However, achieving this strategy necessitated improved access, awareness, and demand generation among the most vulnerable and marginalized groups. Since the project NGOs are deeply entrenched in the project geographies and trusted by the target communities for their health-related needs, they were instrumental in identifying ‘influencers’ from the community, making for an impactful initiative.

Among the transgender community in Chhattisgarh, the team identified and engaged two influencers who were members of the Third Gender Welfare Board constituted by the State Government of Chhattisgarh. Similarly, the team collaborated with a local community in Manipur to create and disseminate socially-inclusive messages and social media posts on vaccine uptake among the targeted marginalized communities of lesbian, gay, bisexual, transgender, intersex, queer/queer-questioning, asexual people (LGBTQI+); people living with HIV/AIDS (PLHIV); individuals who were homeless; orphans; and older adults. The case has been depicted in Box 4.

Box 4 **Countering vaccine hesitancy among transgender people.**

The project team identified the ‘transgender community’ as a sub-group that was vulnerable to COVID-19, but that remained marginalized and could not access vaccination campaigns. The transgender community in the state of Chhattisgarh was hesitant to take the vaccine because many of them were receiving hormone replacement therapy (HRT), and believed that the vaccine would be unsuitable for them. Moreover, with the high prevalence of STDs and the widespread use of HRT, the community believed the myths that vaccination would adversely affect their health due to drug interaction. To increase vaccine uptake among this community, the project team identified a key influencer/ leader from the group who was receptive to vaccination messaging. The team conducted interpersonal counseling sessions with the leader, and developed and showed videos of positive behaviour regarding the safety of vaccines to the community leader. The receptive leader in Chhattisgarh received the first vaccine dose, which motivated the remaining transgender community to take their own vaccinations.

Community engagement was made possible by unconventional partnerships that contributed to improving COVID-19 vaccine uptake. Some of these are detailed below.

The project team engaged ‘Persons with disabilities’ using collaborative efforts and connected them to benefits provided by the social welfare departments. The team also collaborated with NGOs, which organized awareness generation activities by using sign language among teenagers with visual and hearing impairments and cerebral palsy. ‘Drug users’, often situated away from the mainstream, were apprehensive about vaccination due to misconceptions related to the safety of vaccines and fear of side effects. With the support of the Medical Officers at rehabilitation centers, NGOs organized special awareness sessions for drug users and discussed the benefits of the COVID-19 vaccine. The team engaged ‘older adults’; and ‘pregnant and lactating women’ with the help of government systems, including frontline health women workers, Community Health Officers (CHO), Gram Pradhans (village heads), and Panchayat Development Officers (PDO) (who monitor and supervise the work of Gram Pradhans), through counseling and building trust in vaccine safety. The project team mapped ‘tribal villages’ with low COVID-19 vaccination rates in states like Jharkhand, Odisha, Chhattisgarh, and Maharashtra. The project team and NGO staff covered long distances to interact with the communities through folklore activities, in which storytelling was done through acting.

## 4. Results

The project, with its prompt and proactive on-ground implementation, reached approximately 50 million beneficiaries (January–December 2022) through IPC, mid-media (locally-generated communications), hard-copy prints (flyers, leaflets, etc.), and telephonic reminders (on the due date of vaccination and publicizing upcoming camps). The administration of more than 14 million vaccine doses was facilitated between January and December 2022 through ground-based activities by local NGOs and project teams, including mobilizing, facilitating hard-to-reach populations to ongoing camps, and organizing special vaccination camps. Since, the modalities (e.g., folklore, street plays, IPC, communication by faith leaders, community champions, etc.) were culturally acceptable to the target communities, they had a significant contribution in bringing the marginalized communities to the public health mainstream. A significant proportion of doses were facilitated for vulnerable populations (6.1 million). The results are listed activity-wise below.

**Figure 4:**
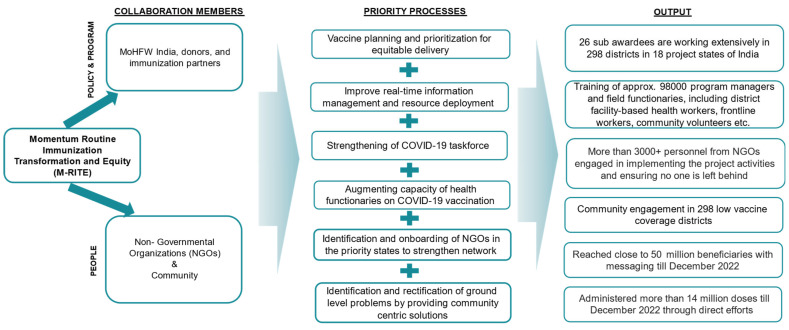
Activities and outcomes.

Figure 4 demonstrates how critical processes based on ground-level insights can bring significant outcomes. The team contributed not only by means of communication, mobilization, and facilitation, but also organized cascade training sessions, and contributed to capacity building of vaccinators, NGOs, and outreach workers.

**Figure 5:**
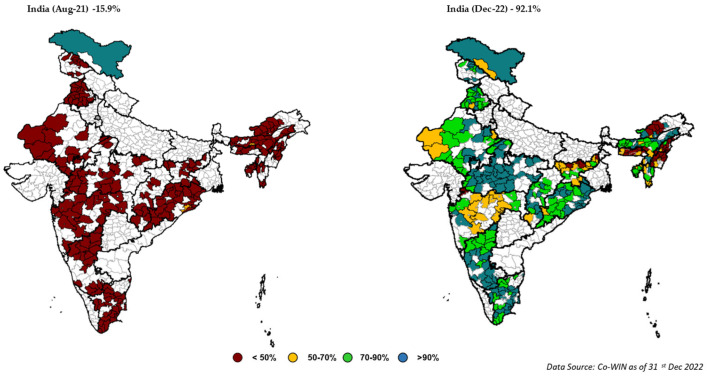
Change in COVID-19 vaccination coverage from August 2021 to January 2023.

Figure 5 showcases the impact of collaborative efforts in the selected 298 districts. The project acted as a catalyst in reaching the eligible population and ensuring vaccination coverage (two-dose coverage) in the target districts. The Government of India’s data (CO-WIN dashboard) showcases that coverage of full immunization coverage (two doses) increased from 15.9% before the commencement of the project in August 2021 to 92.1% by December 2022 in the project intervention states.

**Figure 6:**
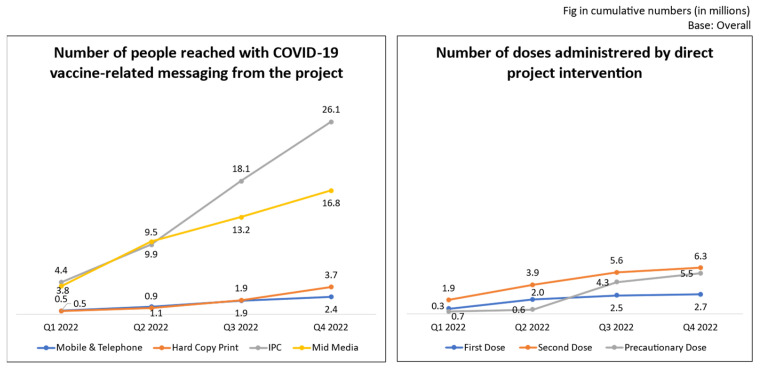
Project efforts in aiding COVID-19 vaccination. Mobile and telephone services included telephonic reminders. Hard-copy printed materials included brochures, IEC communication materials, including printed advertisements in community newspapers, magazines, etc. IPC included one-to-one communication and community meetings, etc. Mid-media include folk performances, street plays, wall paintings, mobile vans containing messages, etc. Telephonic reminders for vaccination were only sent to the people who shared their contact details and verbal consent while attending community engagement, mid-media activities, and vaccination camps for initial doses.

Figure 6 shows the contribution of the project to supporting COVID-19 vaccination in the 18 project states/UTs. Cumulatively, between January 2022 and December 2022, due to direct project initiatives like mobilization and the establishment and support of vaccination camps, over 2.7 million people received the first dose, 6.3 million received the second dose, and 5.5 million received a precautionary dose. Out of the total 14.1 million doses, 6.1 million were given to marginalized and vulnerable individuals, including hard-to-reach, resistant, and neglected groups, such as particularly vulnerable tribal groups (PVTGs), inaccessible working groups (truckers, miners, etc.), sex workers, people with disabilities, transgender people, individuals with specific medical conditions, drug users, etc.

Apart from mobilization and camp facilitation, this success can also be attributed to the IPC efforts of the project. Since the funding mandate prevented the project’s communication activities from using mass media such as social media, TV, radio, etc., the outreach workers utilized innovative communication methods such as folklore, engagement of FBOs, street shows, engagement through community champions etc. Considering the rapid nature of the COVID-19 vaccination initiative and the inaccessibility of the target group, the 14.1 million doses made a significant contribution to reaching the unreached.

**Figure 7:**
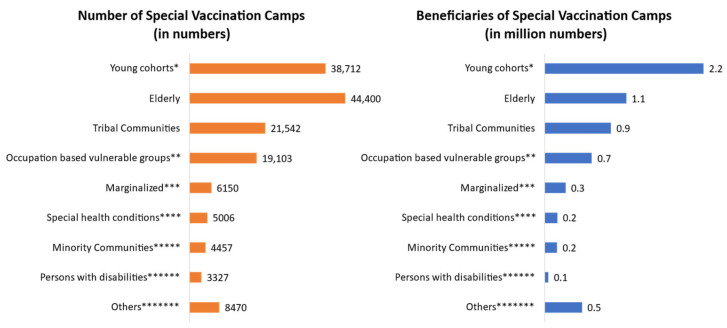
Number of special camps organized for marginalized and vulnerable beneficiaries (until 31 December 2022). * Young cohorts (12–18 years age group) included students, school dropouts, adolescent girls, youth in orphanages, youth in juvenile homes, etc. ** Occupation-based vulnerable groups included workers in factories, brick kilns, salt pans, construction sites, industrial sites, drainage sites, and sugarcane fields, as well as mining workers, daily wagers, truckers, etc.). *** Marginalized included refugees, gypsies, migrants, etc. **** Special health conditions included pregnant and/lactating mothers and people with co-morbidities and other infections. ***** Minority communities included religious minorities such as Sikhs, Muslims, Christians, Buddhists, Jains, etc. ****** Persons with disabilities included mentally and physically challenged people. ******* Others contained in vulnerable groups that were reached in small numbers included jail inmates, drug users, transgender people, etc.

To ensure the fulfillment of one of the key objectives of reaching marginalized and vulnerable population groups, the project team organized special camps to ensure adequate participation of target beneficiaries. Figure 7 provides a detailed account of the numbers achieved at an all-India level. In total, 6.1 million people from marginalized and vulnerable populations benefitted directly from the special vaccination camps organized by the collaboration partners until Dec 2022. State and district-wise information can be obtained on request. The team organized the camps on an as-needed basis, and the number of beneficiaries depended on the population in the nearby areas, hence, the numbers may vary across vulnerable groups.

During the 18 months of project planning and execution, the team gained several learnings regarding the effectiveness of the activities and interventions. State and demographic-wise data can be obtained upon request. The detailed results of MEL activities are outside the purview of this document. The current focus is on highlighting the structured collaborative approach for replication and adaptation in research and practice. The results have been shared briefly to indicate the contribution of the project to COVID-19 vaccination of individuals at an overall and demographic level.

All the approaches to vaccination were instrumental in achieving the project’s objectives. Some activities that were of particular importance were:

- Engagement of government stakeholders: Involving government staff at all levels (State, District, and Block officials) facilitates the timely implementation of interventions (including inter-departmental coordination and conducting vaccination camps) in hard-to-reach areas and catalyzes feedback and action.
- Involvement of local NGOs: Engaging with local NGOs is an effective mechanism for accelerating social and community mobilization. Local NGOs with a strong ground presence and footprint help to quickly map and reach out to target audiences and seek the support of local stakeholders such as village heads, FBOs, self-help groups, elected representatives, etc.
- Contextualization of information, education, and communication (IEC) materials: The development and adaptation of available IEC materials in local languages and local formats ensures quick vaccine acceptance and community action.
- Customized demand generation approaches: Tailored approaches—such as night camps, and collaborations with community and religious leaders to suit local needs and raise awareness of COVID-19 vaccination—help in targeting specific subpopulations that might be more likely to miss vaccinations due to the fear of wage loss and other competing work and family commitments.

## 5. Conclusions

The project’s collaborative approach clearly met the necessary conditions of ‘collaborative rationality’ set forth by Innes and Booher [26]: (1) The collaboration’s participants were diverse, bringing a range of expertise and perspectives to COVID-19 vaccination. (2) The focus was on a topic of interest to all participants. (3) Collaboration participants had high credibility and effectively conducted outreach in their respective communities of interest (the vulnerable and marginalized). (4) The group met face-to-face monthly for a genuine discourse in which all members had equal authority to speak. (5) The group discussed issues and sought strategies to address major community concerns that were shared by each member.

Several considerations were critical to the success of this collaborative approach, including understanding the needs and limitations of the NGOs; working directly with communities to identify appropriate outreach and engagement strategies; prioritizing strategies that supported a multi-directional exchange of information; and adapting strategies based on grounded realities and internal lessons learned. Strategies evolved over time, and the level and quality of community participation increased during planning and implementation. Real-time data analysis facilitated quick and timely decisions; constant effort helped establish collaborations between corporates, funding organizations, and senior team members, which developed mutual confidence because of timely and planned knowledge-sharing sessions. The team overcame many barriers; however, in the process, new obstacles emerged, ones which were then addressed by preemptive approaches. Experiences gained from the project can be used to encourage the consideration of collaborative participation in routine immunization, new vaccine introductions, and other public health programs and decision-making processes.

## 6. Experiences, Limitations, and Potential Considerations

The limitations faced during the project span bring forth certain considerations for community health practices. The challenges, limitations, and lessons from the project’s implementation suggest the following considerations:

### 6.1. Potential Considerations for Practitioners

Public health initiatives targeted at sizable populations are complex due to a combination of behavioral, psychosocial, political, and cultural factors. The conditions at the ground level (particularly in pandemics) require decision-making and the rollout of activities on a war footing. Therefore, it is important to identify and enroll people and agencies who can bring ground-level insights to the initiative and act swiftly to implement changes. Eventually, most program implementation partners enter a collaborative mode, but such collaborations are often unplanned and need-based, therefore, there is a lack of strategic orientation and trustworthiness among partners. Such arrangements often result in overlapping work, non-clarity as to objectives, and fragmentation. The authors propose that a collaborative approach must be at the heart of any vaccination or public health program of this scale. The project activities align with the Immunization Agenda 2030 (IA2030) [27], which emphasizes coordinating the activities of the community, national, regional, and global stakeholders, including governments, regional bodies, global agencies, development partners, and civil society. Based on the project’s limitations, learnings, and experience in convening collaboration, the following checkpoints are recommended for public health professionals and program implementers:

− A strategic orientation with a clearly determined target group and implementation approach is important to keep the activities of collaborators aligned and prevent confusion among the partners.
− Collaboration partners must be chosen wisely through a rigorous vetting process. In the current project, we ensured the recruitment of local Indian NGOs that specialize in dealing with specific vulnerable populations.
− The current project deployed the M&E indicators to keep policy partners informed and aligned with the ground realities on vaccine uptake. However, in the future, the monitoring and evaluation efforts must also be designed according to research priorities.
− Specific requests must be shared with collaborative partners and discussed during follow-up meetings. Achievable plans must be prepared for the next period in tandem with the capacities and potential of collaborative partners.
− Managerial capacities of the collaborating partners (in this case, NGOs) must be upgraded periodically.
− The management of collaborations must aim towards convening rather than commanding and constraining partners. Mutual and respectful cooperation must be fostered by sharing management responsibilities with ground-level partners.
− Community participation by involving religious and community leaders, CBOs, and FBOs, and facilitating a sense of ownership among them must be contemplated in the initiatives targeting marginalized, vulnerable, and hard-to-reach people. The global strategy to ensure maximum inclusion in immunization programs advocates the usage of community-based organizations in strengthening accountability and ownership for immunization at the ground level. The same could be leveraged for reaching out to ‘zero-dose’ vulnerable communities.

### 6.2. Considerations for Researchers in the Future

The need to disseminate research results of public health programs beyond academic and clinical audiences has been expressed repeatedly [28], but limited research output has reached the targeted audience. The dearth of knowledge-sharing is further evident in the area of vaccine service delivery and dissemination. Knowledge sharing would improve the results of subsequent programs. Although the objectives of the current initiative do not include measuring the success of partnerships, this can be an important point of consideration for future research. Similarly, the monitoring efforts which, in the current initiative, were focused on providing timely support for decision-making can be designed to support evaluative research to measure the effectiveness of the intervention. From the current initiative, the project team understood that ‘community engagement’ is an important tool for collecting data from hard-to-reach populations and must be incorporated as a significant instrument in study design, implementation, and evaluation for projects targeted at testing public health concerns.

Overall, the project has devised ways to strengthen the existing healthcare system by identifying and filling existing gaps in equiBox vaccination programming. Efforts to curb vaccine-preventable diseases have, in both the past and the present, recognized the importance of collaborative partnerships which can be utilized to extend routine immunization services to regularly reach “zero dose” and under-immunized children and communities. Engaging community-level partners and ground-based institutions broadens the horizon of public health practice. The local NGOs have a deep understanding of underlying problems in their respective communities. They hold positions of high trustworthiness and receptivity among the communities. The learnings of the project have applicability in the routine immunization program management for the equitable distribution of vaccines by prioritizing efficient storage and redistribution. The coordination between district officials and NGOs was remarkable in this project.

The geographical reach and experiential attributes of these NGOs are well-positioned to be leveraged for reaching the unreached in national primary healthcare programs, with a special focus on routine immunization, strengthening of data systems, appropriate messaging, and emergency response.

## Data Availability

No new data were created or analyzed in this commentary. Data sharing is not applicable to this article.
